# Diagnostic Biopsy Does Not Commonly Induce Intratumoral CD8 T Cell Infiltration in Merkel Cell Carcinoma

**DOI:** 10.1371/journal.pone.0041465

**Published:** 2012-07-31

**Authors:** Shinichi Koba, Kelly G. Paulson, Kotaro Nagase, Andrew Tegeder, Renee Thibodeau, Jayasri G. Iyer, Yutaka Narisawa, Paul Nghiem

**Affiliations:** 1 Dermatology/Medicine, University of Washington, Seattle, Washington, United States of America; 2 Fred Hutchinson Cancer Research Center and Seattle Cancer Care Alliance, Seattle, Washington, United States of America; 3 Division of Dermatology, Department of Internal Medicine, Faculty of Medicine, Saga University, Saga, Japan; The University of Queensland, Australia

## Abstract

**Background:**

Merkel cell carcinoma is a polyomavirus-associated cancer that is strongly linked with T lymphocyte immune suppression in epidemiologic studies. CD8+ T cell infiltration into MCC tumors (intratumoral) has recently been shown to be strongly predictive of improved survival. In contrast, the presence of CD8+ T cells at the border of the tumor (peritumoral) had no independent prognostic value. Spontaneous regression has been reported for MCC approximately one thousand times more often than would be expected given the frequency of this cancer. Many of these events began shortly after biopsy, and in some cases lymphocytic infiltration was described.

**Methodology/Principal Findings:**

To determine whether CD8+ lymphocyte infiltration in MCC tumors is commonly altered by biopsy.33 MCC patients who had microscopic confirmation of MCC on both an initial biopsy and a re-excision specimen were included in this study. Intratumoral and peritumoral CD8 lymphocyte infiltration was quantitated using immunohistochemistry and compared using the paired t-test in biopsy versus re-excision samples. There was a trend toward increased CD8 infiltration after biopsy in a peritumoral (‘stalled’) pattern (p = 0.08), however, biopsy was not associated with a significant increase in CD8 T cells in the clinically more important intratumoral location (p = 0.58).

**Conclusions/Significance:**

The initial diagnostic biopsy for MCC does not commonly alter intratumoral CD8+ T cell infiltration, suggesting it does not directly induce immunologic recognition of this cancer. Because CD8 infiltration is typically stable after biopsy, this parameter may be useful to assess the efficacy of future immune therapies for this virus-associated, immunogenic, often-lethal cancer.

## Introduction

Merkel cell carcinoma (MCC) is an aggressive neuroendocrine skin cancer that often arises on the sun-exposed skin of older individuals with sparse skin pigmentation. The incidence of MCC has tripled over the last 15 years with approximately 1,500 new cases diagnosed in the United States each year. [1], [2]_ENREF_1 MCC has a 46% disease-associated mortality at 5 years [3], significantly higher than that of melanoma (approximately 15%). [4]

A new human polyomavirus (Merkel cell polyomavirus: MCPyV) was detected in 2008 in 80% of MCC tumors. [5] The presence of MCPyV DNA in most MCC tumors has been verified by multiple groups in samples collected from around the world. [5], [6], [7], [8] However, whether or not the tumor’s MCPyV status is associated with survival is controversial. Sihto reported that MCPyV DNA-positive MCC tumors are associated with favorable disease outcome. [9] Touze also described that high titers of antibodies to the VP1 protein of MCPyV were associated with better outcomes. [10] In contrast, Schrama et al. [11] found no improved survival among patients whose tumors were virus-positive.

Although over 90% of MCC patients do not have any clinically apparent immune dysfunction [12], the risk for MCC is markedly increased among persons with chronic T cell dysfunction. Specifically, MCC risk is increased by ∼10-fold after solid organ transplantation [13], by ∼13-fold among HIV-positive patients [14], and by over 30-fold among patients with chronic lymphocytic leukemia. [12], [13], [14] M_ENREF_10oreover, in several case reports, MCC patients experienced tumor regression in response to withdrawal of T cell suppressive therapeutics such as azathioprine [15] or cyclosporine. [16] One HIV+ patient with metastatic MCC experienced a complete and sustained regression of this cancer after initiating highly active antiretroviral therapy together with interleukin-2 administration. [17]

Despite its often-aggressive nature, there are numerous reports of MCC tumors undergoing spontaneous regression (complete resolution of tumor in the absence of any therapy). [18] At least 30 cases of complete spontaneous regression of MCC have been reported. [19] Even though complete spontaneous regression in MCC is described as extremely rare, these cases constitute approximately 1.4% of all reported cases of MCC (15/1,100). [20] Interestingly, once spontaneous regression begins, a full response is typically quite rapid (1 to 5 months) and no instances of recurrence have been reported. [21] Surprisingly, in spite of MCC being more common in men (62%) than women (38%) [3]_ENREF_21, spontaneous regression of MCC is twice as common in women as compared to men. [22] Disease-specific survival is excellent among MCC patients who underwent complete spontaneous regression. Indeed, none are known to have experienced subsequent recurrences. [20], [22] Although the mechanism of spontaneous regression remains unclear, several studies have documented infiltration of T cell lymphocytes (CD3+, CD4+ and CD8+ cells within the tumor) in spontaneous regression of MCC. [23], [24], [25] _ENREF_22_ENREF_23_ENREF_24It is therefore plausible that a T cell mediated immune response is an important event in tumor regression. [26] Furthermore, T-cell-related cytokines such as interferons can promote effective immune responses against neuroendocrine tumors. [27] Most authors who reported spontaneous regression of MCC commented that the diagnostic biopsy could have acted as a trigger of a T cell mediated immune response and subsequent spontaneous regression.

Recently, Paulson et al. [28]_ENREF_28 reported that intratumoral CD8+ lymphocyte infiltration is independently associated with improved MCC-specific survival. In contrast, neither peritumoral CD8+ lymphocyte infiltration nor ‘tumor infiltrating lymphocytes’ as assessed on routine histology were independent predictors of survival. This CD8 assay can readily be carried out on archival paraffin-embedded tissue. We investigated whether the initial biopsy commonly leads to increased intratumoral CD8+ lymphocyte infiltration. We employed both initial biopsy and wide local excision tissue from 33 MCC patients to evaluate intratumoral and peritumoral CD8+ lymphocyte infiltration to test whether biopsy stimulated cellular immunity against MCC.

## Methods

### Ethics Statement

This study was performed in accordance with Helsinki principles and approved by the institutional review board of the Fred Hutchinson Cancer Research Center.

### Patients and Tumors

Patient materials and clinical information used in this study were retrieved from the MCC Tissue and Data Repository at UW. Patients were diagnosed with MCC between 1980 and 2009. MCC diagnosis was confirmed by at least two pathologists. Cases in which the re-excision specimen had no detectable residual Merkel cell carcinoma (and presumably had been fully excised surgically) were not included in this study. Paraffin blocks of biopsy and re-excision tissues from patients enrolled in this Repository were obtained from pathology laboratories, were sectioned and stained in a central study-associated laboratory. MCC stage was determined using 2010 American Joint Committee on Cancer criteria. [3], [29]

Formalin-fixed paraffin-embedded materials of both biopsy and re-excision MCC tissue were available for evaluation from 33 patients. CD8a immunohistochemistry was performed with antibody 4B11 (Novocastra, Bannockburn, IL) as described. [28]_ENREF_28 Tonsil tissue served as positive control and normal mouse serum as negative antibody control.

### CD8 Scoring

CD8 scoring in this study was as described by Paulson et al. [28]_ENREF_28 CD8 scoring was performed by an observer who was blinded as to patient information including outcome. Peritumoral and intratumoral CD8+ cells were scored on a 0 to 5 scale. Peritumoral CD8 cells were defined as CD8+ cells with any stromal contact including intratumoral, neighboring or peritumoral stroma; whereas intratumoral CD8+ cells do not have any direct contact with stroma and are surrounded by tumor cells. The 0–5 score represented average infiltration into the tumor taken as a whole, as opposed to only the densest region of peritumoral and intratumoral infiltration. Figures that represent the various levels of CD8 infiltration are provided in the supplement to Paulson, et al. [28] Ideally, 8–10 representative fields of tumor would be assessed when possible. Effort was made to avoid counting areas with necrosis to minimize false or non-specific reactions.

### Statistical Analysis

Intratumoral and peritumoral CD8 scores from biopsy and re-excision samples were compared with paired t-test. Statistical analysis was performed with Microsoft Excel (Microsoft Corporation, Redmond, WA). Differences were considered statistically significant at p<0.05. STATA software (StataCorp, College Station, TX) was used to generate figures.

## Results

To test whether biopsy induces evidence of effective immune recognition in MCC, we evaluated intratumoral or peritumoral CD8+ lymphocyte infiltration in 33 MCC patients for whom tissue was available from paired biopsy and re-excision specimens. The clinical details of these patients are described and summarized in Table 1 and Table S1. As in other MCC cohorts [3], most patients were male (57%), and most presented with local disease (40% stage I, 30% with stage II). The average interval between initial biopsy and re-excision was 27 days (range 8 to 66). Intratumoral and peritumoral CD8+ cell infiltration was scored separately and semiquantitatively as described. [28] Although 33 patients were scored for intratumoral CD8+ cells, only 31 patients could be scored for peritumoral CD8+ cells because two biopsies had insufficient peritumoral tissue for evaluation.

**Table 1:** Clinical and tumor characteristics among 33 study subjects.

Characteristic	No. (%)
**Age at diagnosis, years**	
Mean	70
Range	48–91
**Male sex**	19 (57)
Stage at MCC presentation*	
I	13 (40)
II	10 (30)
III	9 (27)
IV	1 (3)
**Interval between biopsy and re-excision, days**	
Mean	27
Range	8–66

Stage was determined as per AJCC 2010 criteria.

**Figure 1:**
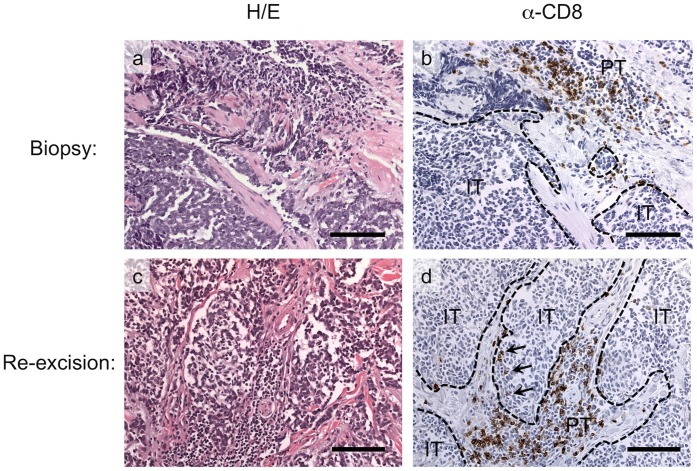
Peritumoral and intratumoral CD8 lymphocyte infiltration at biopsy and re-excision of a representative MCC tumor. Top row: Biopsy specimen stained with H/E (a) and á-CD8 (b) from case # w313. Bottom row: Re-excision section stained with H/E (c) and á-CD8 (d) from the same case. Peritumoral and intratumoral CD8 lymphocytic infiltrates were each scored on a 0 to 5 scale as described. These images correspond to peritumoral CD8 score of 3 and intratumoral CD8 score of 0 at biopsy, and peritumoral CD8 score of 4 and intratumoral CD8 score of 1 at re-excision. CD8 scores represent average peri-/intra-tumoral infiltration across many more fields than are visible in the figure. Importantly, CD8 cells that are in contact with stroma are not considered to be intratumoral. [28] The black dashed lines in á-CD8 images indicate the tumor/stromal interface (intratumoral: IT, peritumoral: PT). Brown cells indicated by arrows in panel d) are CD8+ cells in a true intra-tumoral location. Scalebar 100 uM. Abbreviations: H/E, hematoxylin and eosin.

Histologic characteristics of a representative patient are shown in Figure 1. This patient was a 77 year-old man who presented with a 4.8 cm MCC on the left temple with radiologically apparent regional lymph node involvement (stage IIIb). Although peritumoral CD8+ cells increased modestly between the time of biopsy (2 on scale of 0–5) to re-excision (score of 4), the intratumoral CD8 infiltrate was stable and very modest (score of 1) on this scale at the time of biopsy and re-excision.

**Figure 2:**
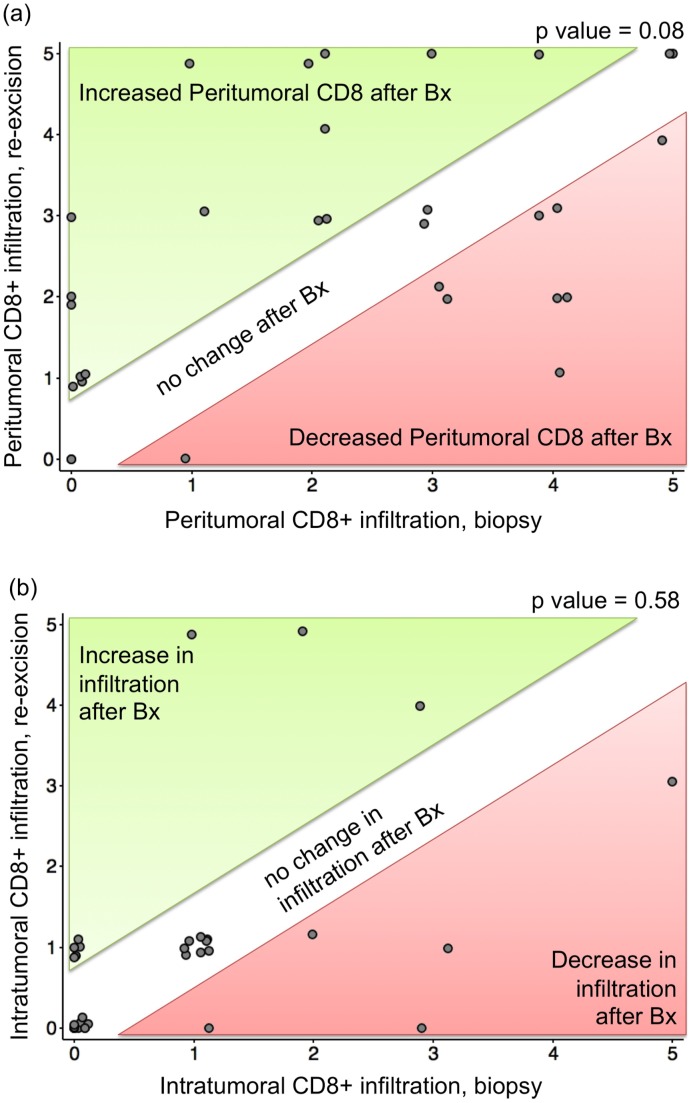
CD8 cell infiltration of paired biopsy and re-excision specimens. Each circle represents a pair of specimens from an MCC patient. **Fig. 2a** shows the extent of peritumoral CD8 infiltration (n = 31), and **Fig. 2b** represents intratumoral CD8 infiltration (n = 33; two cases had insufficient tissue to evaluate peritumoral infiltration). Cases for which the two specimens were identical in CD8 infiltration are depicted along the diagonal that is not shaded (marked with “no change after Bx”). Cases that had an increase in infiltration after biopsy are in the top left (green shaded) area. Cases with a decrease in infiltration after biopsy are in the lower right (red shaded) area. Abbreviations: Bx, biopsy. There was no statistically significant difference in CD8 infiltration for either location, intratumoral (P-value = 0.08) or peritumoral (P-value = 0.58) via paired-t test.

Among the 31 tumors evaluable for peritumoral CD8 infiltration, the mean peritumoral score at biopsy on the 6-point scale was 2.2 compared to a re-excision score of 2.7 (p = 0.08; paired t-test) (Figure 2a). Thus, although there was a small trend toward increased CD8 T cell infiltration in a peritumoral (‘stalled’) pattern in re-excision samples, this trend did not reach statistical significance. Importantly, intratumoral CD8 infiltration scores among 33 evaluable cases were essentially stable with a mean score at biopsy of 0.8 compared to 1.0 at re-excision (p = 0.58; paired t-test) (Figure 2b). Biopsy was thus not associated with an increase in the biologically more important intratumoral CD8 T cells.

## Discussion

Spontaneous regression of MCC has been reported far more frequently than would be expected for a cancer of this incidence, despite its often-aggressive nature. The T cell mediated immune response is likely an important event in spontaneous regression of MCC, and the initial diagnostic biopsy has been suggested as a trigger of an effective anti-tumor immune response. It was recently reported that intratumoral CD8+ lymphocyte infiltration is strongly associated with improved MCC-specific survival. [28] Moreover, among patients with good outcome, unbiased gene expression analyses showed overexpression of CD8 lymphocyte-associated genes including granzymes, chemokines (CCL19), lymphocyte-activation molecules, and CD8 receptor molecules. [28] CCL19 is an inflammatory chemokine that can promote inflammatory responses. [30], [31] It is plausible that a surgical procedure such as a biopsy might induce CCL19 and hence promote T cell infiltration. Recently, Sihto et al. also reported that intratumoral CD3+ (and CD8+) cell infiltration was associated with improved overall survival in a Finnish MCC cohort. [32] We therefore evaluated whether CD8+ lymphocyte infiltration in MCC tumors is commonly altered by biopsy. In the present study, we found there was no statistically significant increase in intratumoral CD8+ cell infiltration following biopsy in paired specimens. Biopsy also did not significantly induce inflammation in terms of peritumoral CD8+ cells. Our data suggest that biopsy does not commonly induce immunologic recognition of this cancer.

In other malignancies, spontaneous regression is an extremely rare phenomenon. It has been documented in several types of malignancies, such as malignant melanoma [33], neuroblastoma [34], lung cancer [35], renal cell carcinoma [36], [37], hepatocellular carcinoma [38], [39], colorectal cancer [40], and lymphoma [41], [42]. Based on reports in the literature, this occurs less than once in 60,000 to 100,000 people with cancer, with about 20 such cases reported annually among all other cancers [43]. Mechanisms and triggers of spontaneous regression have been hypothesized to include infection, pregnancy, and surgical procedures such as biopsy. These processes may underlie the induction of a relevant immune response involving lymphocyte infiltration into the tumor and recognition of specific antigens. [44] In the rare spontaneous regression of other malignancies, CD8+ T cells appear to be an important effector mechanism of anti-tumor immunity. [45] The presence of tumor-infiltrating lymphocytes (TILs) was associated with improved disease outcome in various other tumors, such as esophageal, ovarian, renal, and colon carcinoma. [46], [47], [48], [49], [50], [51] Stage IV non-small cell lung cancer patients with more tumor-infiltrating CD8+ T cells in cancer nests (intratumoral) than in cancer stroma showed significantly better survival. [52] Peritumoral lymphocytes were not related to outcome in other cancers, such as ovarian cancer and colon cancer. [46], [53]

There are several limitations of this study. (1) The number of MCC cases with appropriate before/after specimens (n = 33 for intratumoral lymphocyte evaluation) is limited; (2) Complete spontaneous regression is relatively uncommon even in MCC; (3) If no tumor was present at the time of re-excision, the case could not be evaluated for ‘intratumoral’ infiltration of CD8+ cells. (4) In our study, the median age of the MCC patient population was slightly younger (70 years as compared to 76 years). [3] Despite these limitations, it is clear from these data that biopsy does not commonly induce an immune infiltrate associated with improved survival in MCC.

The present study may be useful for determining the efficacy of future immune therapeutics in this cancer. Given the documented relevance of immune function in surviving after an MCC diagnosis, it is likely there will be multiple future trials of immune-stimulating agents in this cancer. If a novel treatment for MCC is found to induce a CD8+ lymphocyte infiltrate into the tumor, it is likely that it is causing a direct/desired immune effect rather than merely a post-surgical inflammatory effect. For example, intralesional â-interferon was reported to be effective in treating an MCC patient with multiple metastases, although that study did not examine effects on immune infiltration. [54] It is plausible that intralesional interferon therapy induced CD8+ cell infiltration as part of its mechanism that led to a complete durable response that at last followup had persisted for eight years. In the future, MCPyV-targeted immunotherapies may be developed, based in part on recent insights into the T cell immune response against this virus [55]. Clinical trials of blockers of the PD-1 pathway and of CTLA4 are also likely to be carried out. In each of these cases, if CD8+ lymphocytes are recruited into the tumor, the present study suggests that this is likely due to the novel therapeutic agent rather than to post-biopsy inflammatory changes.

Although it has been suggested that biopsy of MCC may induce an immune response, this study showed that this minor surgical procedure does not frequently induce a validated hallmark of cell mediated immunity against MCC: CD8 intratumoral infiltration. For this reason, this marker that can be readily assessed on archival/baseline tissue, may be useful in determining whether or not an intervention under study is promoting an effective immune response against this often-lethal skin malignancy.

## Supporting Information

Table S1 **The detailed information of 33 study subjects.** W# is patient’s number in the University of Washington Data repository of this study. Peritumoral score of W# 197 and 248 were not available. Abbreviations: M, male; F, female; Bx, biopsy; Peri, peritumoral; Intra, intratumoral; N.S, not scored.
(DOC)
