# Correction: Diagnostic Biopsy Does Not Commonly Induce Intratumoral CD8 T Cell Infiltration in Merkel Cell Carcinoma

**DOI:** 10.1371/annotation/c4df91b0-618f-464d-a613-d99758de3f3d

**Published:** 2012-10-16

**Authors:** Shinichi Koba, Kelly G. Paulson, Kotaro Nagase, Andrew Tegeder, Renee Thibodeau, Jayasri G. Iyer, Yutaka Narisawa, Paul Nghiem

There were multiple errors throughout the article.

There was a formatting error that inserted the text "_ENREF_#" in numerous occasions throughout the article. These insertions should not exist.

In the Figure 1 legend, the symbol á should be α.

In the fourth paragraph of the Discussion section, the symbol "â" in the term "â-interferon" should be "beta," making the term "beta-interferon."

