# Supplementary material for: Diagnostic Biopsy Does Not Commonly Induce Intratumoral CD8 T Cell Infiltration in Merkel Cell Carcinoma
Source: PLoS One. 2012 Jul 31;7(7):e41465. doi: 10.1371/journal.pone.0041465 (PMC3409202; doi:10.1371/journal.pone.0041465)
Supplement: Table S1 — The detailed information of 33 study subjects. W# is patient’s number in the University of Washington Data repository of this study. Peritumoral score of W# 197 and 248 were not available. Abbreviations: M, male; F, female; Bx, biopsy; Peri, peritumoral; Intra, intratumoral; N.S, not scored. (DOC) [file pone.0041465.s001.doc]

Table S1.

|  |  |  |  |  |  | Days | CD8 infiltration | | | | | | |
| --- | --- | --- | --- | --- | --- | --- | --- | --- | --- | --- | --- | --- | --- |
| W# | Sex | Age | Site on | Tumor | Stage | Between | Bx | | |  | Re-excision | | |
|  |  |  | Body | Size |  | Specimens | Peri |  | Intra |  | Peri |  | Intra |
| 58 | F | 66 | upper limb | 1 | 1 | 22 | 0 |  | 0 |  | 2 |  | 0 |
| 74 | M | 77 | head & neck | 0.5 | 1 | 41 | 0 |  | 0 |  | 1 |  | 0 |
| 145 | M | 66 | head & neck | 1 | 1 | 8 | 0 |  | 0 |  | 1 |  | 0 |
| 156 | F | 65 | upper limb | 2 | 3 | 38 | 1 |  | 1 |  | 5 |  | 5 |
| 170 | M | 82 | upper limb | 1 | 1 | 15 | 0 |  | 0 |  | 2 |  | 0 |
| 197 | M | 87 | head & neck | 3.2 | 2 | 16 | N.S |  | 1 |  | 4 |  | 1 |
| 200 | M | 60 | upper limb | 9 | 4 | 29 | 2 |  | 2 |  | 3 |  | 1 |
| 209 | M | 49 | upper limb | 2.5 | 2 | 21 | 3 |  | 3 |  | 5 |  | 4 |
| 216 | F | 77 | buttock | 1 | 1 | 48 | 4 |  | 3 |  | 5 |  | 0 |
| 224 | F | 74 | upper limb | 5.5 | 3 | 47 | 5 |  | 1 |  | 4 |  | 1 |
| 229 | F | 70 | upper limb | 4 | 2 | 13 | 2 |  | 5 |  | 5 |  | 3 |
| 235 | M | 71 | lower limb | 5.6 | 3 | 32 | 1 |  | 0 |  | 3 |  | 0 |
| 238 | M | 48 | lower limb | 7 | 2 | 8 | 4 |  | 1 |  | 2 |  | 1 |
| 239 | M | 68 | head & neck | 0.5 | 3 | 35 | 2 |  | 0 |  | 3 |  | 1 |
| 244 | F | 58 | head & neck | 0.9 | 1 | 53 | 5 |  | 0 |  | 5 |  | 1 |
| 248 | M | 50 | trunk | 5.3 | 2 | 18 | N.S |  | 0 |  | 3 |  | 1 |
| 249 | F | 68 | head & neck | 0.59 | 1 | 19 | 0 |  | 0 |  | 1 |  | 0 |
| 251 | M | 73 | upper limb | 4 | 3 | 46 | 2 |  | 1 |  | 5 |  | 1 |
| 252 | M | 72 | head & neck | 1.1 | 3 | 20 | 4 |  | 2 |  | 1 |  | 5 |
| 255 | F | 86 | trunk | 2.2 | 2 | 13 | 3 |  | 1 |  | 3 |  | 0 |
| 264 | M | 70 | trunk | 7 | 2 | 37 | 0 |  | 0 |  | 0 |  | 0 |
| 269 | F | 64 | trunk | 2 | 3 | 12 | 3 |  | 0 |  | 2 |  | 0 |
| 273 | F | 82 | upper limb | 0.8 | 1 | 23 | 0 |  | 0 |  | 1 |  | 0 |
| 282 | M | 81 | lower limb | 0.8 | 1 | 19 | 0 |  | 0 |  | 0 |  | 1 |
| 297 | M | 77 | head & neck | 0.3 | 1 | 28 | 1 |  | 0 |  | 0 |  | 0 |
| 298 | M | 64 | head & neck | 6 | 2 | 66 | 4 |  | 3 |  | 3 |  | 1 |
| 302 | F | 52 | head & neck | 1.3 | 1 | 15 | 5 |  | 1 |  | 5 |  | 1 |
| 309 | M | 68 | upper limb | 0.8 | 1 | 45 | 4 |  | 0 |  | 2 |  | 1 |
| 313 | M | 77 | head & neck | 4.8 | 3 | 10 | 2 |  | 1 |  | 4 |  | 1 |
| 337 | M | 91 | lower limb | 3 | 3 | 27 | 4 |  | 1 |  | 3 |  | 1 |
| 347 | F | 78 | upper limb | 3.5 | 2 | 22 | 3 |  | 1 |  | 3 |  | 1 |
| 349 | F | 74 | upper limb | 1 | 2 | 26 | 0 |  | 0 |  | 3 |  | 0 |
| 363 | F | 71 | head & neck | 0.8 | 1 | 21 | 3 |  | 0 |  | 2 |  | 0 |
